# Let’s talk about it: An atypical case

**DOI:** 10.1192/j.eurpsy.2021.1714

**Published:** 2021-08-13

**Authors:** A. Gonzaga Ramírez, Ó. Martín, G. Guerra Valera, M. Queipo De Llano De La Viuda

**Affiliations:** Psiquiatría, Hospital Clínico Universitario de Valladolid, Valladolid, Spain

**Keywords:** alcohol use disorder, eating disorders, Addiction, Dual pathology

## Abstract

**Introduction:**

Cross-sectional studies report the high comorbility of substance use (SUD) with eating disorders (ED). This case report aims to describe a case of anorexia nervosa and alcohol use disorder in a 18 year old male.

**Objectives:**

Based on the need to formulate protocols, we aim to conduct a systematic review on the recent literature research on this coexisting psychiatric disorders.

**Methods:**

Relevant studies were sourced from published literature and reviewed.

**Results:**

The prevalence of ED is higher in women than in men, with a ratio of 7:1; however it is the latter that present the most serious clinical pictures. It should be also noted that no all types of ED present the same comorbility, but rather those with bulimic symptoms are the ones that most resort to substance abuse, so the distinction between subtypes is highly relevant.

**Conclusions:**

It is important that clinicians are aware of the severity of this combination and the need for a specific and careful management. Also important to taking into account the limited bibliography on the subject, it is especially important to expand research.

**Disclosure:**

No significant relationships.

